# A Cross-Sectional Analysis of the Readability of Online Information Regarding Hip Osteoarthritis

**DOI:** 10.7759/cureus.60536

**Published:** 2024-05-18

**Authors:** Brandon Lim, Ariel Chai, Mohamed Shaalan

**Affiliations:** 1 Department of Medicine, School of Medicine, Trinity College Dublin, Dublin, IRL; 2 Department of Orthopaedics and Traumatology, The Mater Misericordiae University Hospital, Dublin, IRL; 3 Department of Trauma and Orthopaedics, St James's Hospital, Dublin, IRL

**Keywords:** online medical education, orthopaedic surgery, healthcare education, internet, hip osteoarthritis

## Abstract

Introduction

Osteoarthritis (OA) is an age-related degenerative joint disease. There is a 25% risk of symptomatic hip OA in patients who live up to 85 years of age. It can impair a person’s daily activities and increase their reliance on healthcare services. It is primarily managed with education, weight loss and exercise, supplemented with pharmacological interventions. Poor health literacy is associated with negative treatment outcomes and patient dissatisfaction. A literature search found there are no previously published studies examining the readability of online information about hip OA.

Objectives

To assess the readability of healthcare websites regarding hip OA.

Methods

The terms “hip pain”, “hip osteoarthritis”, “hip arthritis”, and “hip OA” were searched on Google and Bing. Of 240 websites initially considered, 74 unique websites underwent evaluation using the WebFX online readability software (WebFX®, Harrisburg, USA). Readability was determined using the Flesch Reading Ease Score (FRES), Flesch-Kincaid Reading Grade Level (FKGL), Gunning Fog Index (GFI), Simple Measure of Gobbledygook (SMOG), Coleman-Liau Index (CLI), and Automated Readability Index (ARI). In line with recommended guidelines and previous studies, FRES >65 or a grade level score of sixth grade and under was considered acceptable.

Results

The average FRES was 56.74±8.18 (range 29.5-79.4). Only nine (12.16%) websites had a FRES score >65. The average FKGL score was 7.62±1.69 (range 4.2-12.9). Only seven (9.46%) websites were written at or below a sixth-grade level according to the FKGL score. The average GFI score was 9.20±2.09 (range 5.6-16.5). Only one (1.35%) website was written at or below a sixth-grade level according to the GFI score. The average SMOG score was 7.29±1.41 (range 5.4-12.0). Only eight (10.81%) websites were written at or below a sixth-grade level according to the SMOG score. The average CLI score was 13.86±1.75 (range 9.6-19.7). All 36 websites were written above a sixth-grade level according to the CLI score. The average ARI score was 6.91±2.06 (range 3.1-14.0). Twenty-eight (37.84%) websites were written at or below a sixth-grade level according to the ARI score.

One-sample t-tests showed that FRES (p<0.001, CI -10.2 to -6.37), FKGL (p<0.001, CI 1.23 to 2.01), GFI (p<0.001, CI 2.72 to 3.69), SMOG (p<0.001, CI 0.97 to 1.62), CLI (p<0.001, CI 7.46 to 8.27), and ARI (p<0.001, CI 0.43 to 1.39) scores were significantly different from the accepted standard.

One-way analysis of variance (ANOVA) testing of FRES scores (p=0.009) and CLI scores (p=0.009) showed a significant difference between categories. Post hoc testing showed a significant difference between academic and non-profit categories for FRES scores (p=0.010, CI -15.17 to -1.47) and CLI scores (p=0.008, CI 0.35 to 3.29).

Conclusions

Most websites regarding hip OA are written above recommended reading levels, hence exceeding the comprehension levels of the average patient. Readability of these resources must be improved to improve patient access to online healthcare information which can lead to improved patient understanding of their own condition and treatment outcomes.

## Introduction

Osteoarthritis (OA) is an age-related degenerative joint disease that affects both the articular cartilage and surrounding tissues [1]. The hip is a large weight-bearing joint that can commonly develop OA via a process which involves progressive loss of articular cartilage, subchondral cysts, osteophyte formation, periarticular ligamentous laxity, muscle weakness, and synovial inflammation [1-3]. People who live to 85 years of age have a 25% lifetime risk of developing symptomatic hip OA [2]. Hip OA reduces mobility and independence and increases disability and dependency in daily activities which can lead to increased reliance on healthcare services [1]. Hip OA is conservatively managed using prescribed exercise, weight loss, and education which can be further complemented with non-steroidal anti-inflammatory drugs, intra-articular steroid injections, and duloxetine while a total joint replacement may be indicated in severe OA [1-3].

Health literacy is defined as “the ability of an individual to obtain and translate knowledge and information to maintain and improve health in a way that is appropriate to the individual and system contexts” [4]. Personal knowledge about a disease is a key component of health literacy and has a significant impact on treatment outcomes [5]. Poor health literacy is associated with poor outcomes due to a reduced understanding of instructions [5]. Examples of poor outcomes include re-presentations to the hospital, increased inpatient stay, increased post-operative morbidity and mortality, and reduced patient satisfaction [6-9]. In the United States, around 32 million American adults are illiterate and 68 million read below a fifth-grade level [10]. To address this issue, organisations such as The National Institutes of Health (NIH) and the American Medical Association (AMA), and previously conducted studies regarding the readability of online healthcare resources, have recommended that online information directed at patients should be written at or below a sixth-grade level to be deemed acceptable for the public [6,10,11].

We conducted an extensive literature search and have not found any previously published study examining the readability of online information about hip OA. There were similar articles conducted regarding the readability of online resources regarding OA in general [12], OA of the knees [13-16] and hands [17]. There was one recently published study evaluating the quality of online resources regarding hip OA but not the readability of online resources [18]. Therefore, to fill this gap in the literature, this study aims to carry out a cross-sectional evaluation of the readability of healthcare information on the internet regarding hip OA.

## Materials and methods

Search strategy

In April 2024, websites with patient information regarding hip OA were identified using Google and Bing, the two largest search engines by market share at the time of this investigation [19]. Cookies, location, and user account information were disabled before each search to avoid any unintended bias in search results. Search terms were identified using Google Trends. Search terms used ranged from lower complexity terms (“hip pain”) to higher complexity terms (“hip osteoarthritis”, “hip arthritis”, and “hip OA”), resulting in a total of eight unique searches. Table 1 shows the number of hits returned from each search engine and search term combination.

**Table 1:** Hits returned for each search engine and search term combination. OA: osteoarthritis

Search engine	Hits returned
Google & hip pain	1,050,000,000
Google & hip osteoarthritis	156,000,000
Google & hip arthritis	217,000,000
Google & hip OA	27,900,000
Bing & hip pain	369,000
Bing & hip osteoarthritis	192,000
Bing & hip arthritis	243,000
Bing & hip OA	125,000

**Figure 1:**
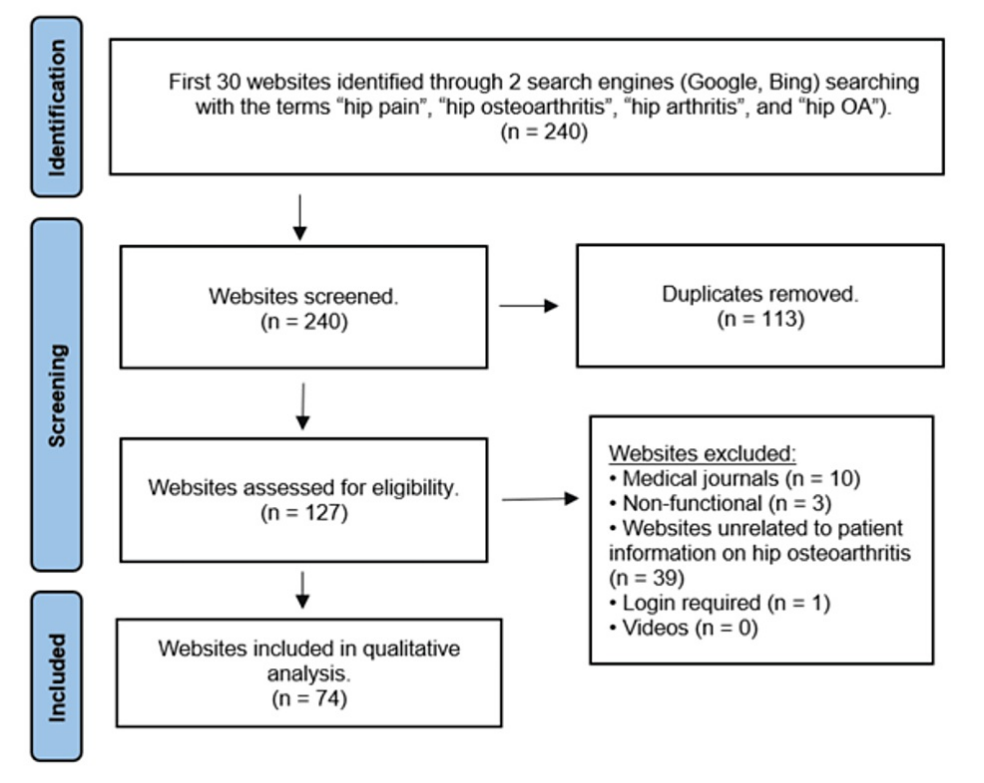
Flow diagram of the methodology used for screening websites (based on the Preferred Reporting Items for Systematic Reviews and Meta-Analyses statement). OA: osteoarthritis

The first 30 results from each of the eight unique searches, a total of 240 websites, were screened. This limitation was set according to search strategies utilised by previous studies that showed that most people do not look beyond the first two to three pages of results on a search engine [6,20]. Non-functional websites, duplicate websites, websites unrelated to patient information regarding hip OA, websites requiring logins, YouTube videos, and websites composed solely of videos were excluded. Medical journals were excluded in concordance with previous studies that found their complexity beyond the understanding of the general population [6,21]. This methodology is concordant with similar studies previously published in the literature [6,20]. Figure 1 illustrates a breakdown of this methodology.

Websites chosen for analysis were further categorised according to methodologies established in previously published studies [14,22,23]. Categories include (1) "commercial" websites referring to websites affiliated with commercial organizations that act as sources of health information; (2) "academic" websites referring to websites affiliated with universities, academic hospitals, and academic societies; (3) "non-academic" websites referring to websites associated local hospitals and private practices; (4) "non-profit" websites referring to those maintained by a national government or government organizations or a non-for-profit organization; and (5) "miscellaneous" websites referring to websites that do not fit the aforementioned four categories. All websites were reviewed by two independent authors within one week of the original search.

Analysis of readability

Websites were uploaded onto an online readability software called WebFX (WebFX®, Harrisburg, USA) [24]. Readability was assessed using six validated algorithms for readability which determined readability according to the number of characters or syllables per word: (1) the Flesch Reading Ease Score (FRES); (2) the Flesch-Kincaid Reading Grade Level (FKGL); (3) the Gunning Fog Index (GFI); (4) the Simple Measure of Gobbledygook (SMOG); (5) the Coleman-Liau Index (CLI); and (6) the Automated Readability Index (ARI). Scores are summarised in Table 2. A detailed breakdown of the FRES can be found in Table 3. Online healthcare-related resources written at or below a US sixth-grade level were considered acceptable [6,11,21,25]. A FRES score of at least 65 is acceptable for public reading standards [6,21,25].

**Table 2:** Summary of readability scores ASL: number words/number sentences; ASW: number syllables divided by number of words; C**: complex words with exceptions including, proper nouns, words made of three syllables by addition of "ed" or "es", compound words made of simpler words; C: complex words (≥3 syllables); L: average number of letters per 100 words; N: number of syllables; S: average number of sentences per 100 words; W: number of words

Score	Scoring basis	Formula
Flesch Reading Ease Score (FRES)	Index score from 0 to 100.	(206.835 - (84.6 × ASW) - (1.015 × ASL)
Flesch-Kincaid Reading Grade Level (FKGL)	Grade level.	(11.8 × ASW) + (0.39 × ASL) -15.59
Gunning Fog Index (GFI)	Grade level.	0.4 × (ASL+((C**/W) × 100))
Simple Measure of Gobbledygook (SMOG)	Grade level.	1.0430 × √C+3.1291
Coleman-Liau Index (CLI)	Grade level.	0.0588L-0.296S-15.8
Automated Readability Index (ARI)	Grade level.	4.71 (characters/words)+0.5 (ASL) - 21.43

**Table 3:** Summary of the Flesch Reading Ease Score

Score	Level of education	Description
90-100	5th Grade	Very easy to read. Easily understood by an average 11-year-old student.
80-90	6th Grade	Easy to read. Conversational English for consumers.
70-80	7th Grade	Fairly easy to read.
60-70	8th to 9th Grade	Plain English. Easily understood by 13-year to 15-year-old students.
50-60	10th to 12th Grade	Fairly difficult to read.
30-50	College	Difficult to read.
0-30	College Graduate	Very difficult to read. Best understood by university graduates.

Statistical analysis

Statistical analysis was performed using Statistical Package for the Social Sciences (IBM SPSS Statistics for Windows, IBM Corp., Version 29.0, Armonk, NY) [26]. P values <0.05 were deemed significant. Analysis of variance (ANOVA) testing was performed to determine the difference between categories. If ANOVA testing achieved significance, post-hoc statistics using Tukey’s test were then undertaken. One-sample t-tests were used to compare FRES scores with the recommended standard of 65, while grade-level scores were compared with the recommended sixth-grade standard.

Ethical approval

Patients and the public were not involved in this study. This internet-based study without human subjects did not require institutional review board approval.

## Results

Reading levels

A total of 74 websites underwent qualitative analysis. Table 4 shows a list of included websites from both search engines (Google and Bing). Among these, there were 17 commercial websites, 19 academic websites, 12 non-academic medical websites, 20 non-profit websites, and six miscellaneous websites. Readability scores by FRES, FKGL, GFI, SMOG, CLI, and ARI for all websites were analysed and are shown in Table 5.

**Table 4:** List of websites by search engine

Search engine	Websites
Google	https://versusarthritis.org/about-arthritis/conditions/hip-pain/ https://www.webmd.com/pain-management/hip-pain-causes-and-treatment https://www.nhs.uk/conditions/hip-pain/ https://my.clevelandclinic.org/health/symptoms/21118-hip-pain https://www.healthline.com/health/hip-pain https://www.pennmedicine.org/for-patients-and-visitors/patient-information/conditions-treated-a-to-z/hip-pain https://www.nidirect.gov.uk/conditions/hip-pain-adults https://www.rush.edu/news/7-common-causes-hip-pain https://www.hss.edu/condition-list_hip-pain-causes.asp https://www.arthritis.org/health-wellness/about-arthritis/where-it-hurts/when-hip-pain-may-mean-arthritis https://www.arthritis-health.com/blog/whats-causing-my-hip-pain https://www.healthdirect.gov.au/hip-pain https://www.webmd.com/arthritis/ss/slideshow-hip-pain-causes https://alexanderorthopaedics.com/blog/why-does-my-hip-hurt/ https://www.aurorahealthcare.org/services/orthopedics/conditions/hip-pain https://www.hss.edu/article_hip-pain-when-walking.asp https://www.tims.nhs.uk/self-care/hip/ https://www.hopkinsmedicine.org/health/conditions-and-diseases/hip-arthritis# https://orthoinfo.aaos.org/en/diseases--conditions/osteoarthritis-of-the-hip/ https://versusarthritis.org/about-arthritis/conditions/osteoarthritis-oa-of-the-hip/ https://www.webmd.com/osteoarthritis/hip-osteoarthritis-degenerative-arthritis-hip https://www.ortho.wustl.edu/content/Patient-Care/3207/Services/Hip-Knee/Adult-Reconstruction-and-Hip-Preservation-Overview/Arthritis-of-the-Hip.aspx https://sportssurgeryclinic.com/services/hip-surgery/hip-osteoarthritis/ https://www.mayoclinic.org/diseases-conditions/osteoarthritis/symptoms-causes/syc-20351925 https://orthop.washington.edu/patient-care/articles/hip/osteoarthritis-of-the-hip-hip-arthritis.html https://www.england.nhs.uk/wp-content/uploads/2022/07/Making-a-decision-about-hip-osteoarthritis.pdf https://www.arthritis.org/health-wellness/about-arthritis/understanding-arthritis/hip-osteoarthritis https://www.arthritis-health.com/video/hip-osteoarthritis-video https://www.wihb.scot.nhs.uk/wp-content/uploads/2020/03/Hip-Osteoarthritis-Information.pdf https://www.arthritis-health.com/types/osteoarthritis/hip-osteoarthritis-symptoms-and-signs https://www.hss.edu/condition-list_hip-arthritis.asp https://www.healthline.com/health/osteoarthritis/hip-treatments https://www.arthritis-health.com/types/osteoarthritis/what-hip-osteoarthritis https://www.brighamandwomens.org/orthopaedic-surgery/osteoarthritis-of-the-hip https://www.medicalnewstoday.com/articles/327023 https://www.nhslanarkshire.scot.nhs.uk/services/physiotherapy-msk/hip-osteoarthritis/ https://www.nhs.uk/conditions/osteoarthritis/ https://www.aberdeenorthopaedics.com/hip-osteoarthritis-the-four-stages/ https://www.arthritis-health.com/video/symptoms-hip-osteoarthritis-video https://www.cedars-sinai.org/health-library/diseases-and-conditions/a/arthritis-of-the-hip.html https://www.hrorthopaedics.co.uk/hips/hip-arthritis/ https://www.arthritis-health.com/blog/my-hip-pain-arthritis-or-bursitis https://www.gleneagles.com.sg/health-plus/article/hip-arthritis-symptoms-treatment https://sportsmedicine.mayoclinic.org/condition/early-mild-arthritis/ https://www.beaumont.org/conditions/hip-arthritis https://creakyjoints.org/living-with-arthritis/symptoms/arthritis-in-hips/ https://orthoinfo.aaos.org/globalassets/pdfs/hip-osteoarthritis.pdf https://www.versusarthritis.org/media/22728/osteoarthritis-of-the-hip-information-booklet.pdf https://complete-physio.co.uk/osteoarthritis-oa-of-the-hip/
Bing	https://www.emedicinehealth.com/hip_pain/article_em.htm https://www.medicalnewstoday.com/articles/hip-pain-when-walking https://www.verywellhealth.com/injuries-and-conditions-causing-hip-pain-2548630 https://www.healthline.com/health/pain-relief/exercises-for-hip-pain https://www.mayoclinic.org/symptoms/hip-pain/basics/causes/SYM-20050684?p=1 https://en.wikipedia.org/wiki/Hip_pain https://www.medicalnewstoday.com/articles/hip-pain-when-sitting https://www.mayoclinic.org/diseases-conditions/osteoarthritis/diagnosis-treatment/drc-20351930 https://www.nhs.uk/conditions/osteoarthritis/symptoms/ https://www.medicalnewstoday.com/articles/stages-of-osteoarthritis-of-the-hip https://orthoinfo.aaos.org/en/diseases--conditions/osteoarthritis https://www.arthritisireland.ie/osteoarthritis https://my.clevelandclinic.org/health/diseases/5599-osteoarthritis https://www.ucsfhealth.org/conditions/osteoarthritis-of-the-hip https://www.arthritis.org.nz/osteoarthritis/ https://www.nhs.uk/conditions/osteoarthritis/treatment/ https://www.verywellhealth.com/hip-arthritis-2548627 https://www.arthritis-health.com/types/osteoarthritis/hip-osteoarthritis-causes-and-risk-factors https://www.verywellhealth.com/stages-of-osteoarthritis-of-the-hip-5093151 https://www.verywellhealth.com/symptoms-of-arthritis-in-hips-5104650 https://www.mayoclinic.org/diseases-conditions/arthritis/in-depth/arthritis/art-20046440 https://www.mayoclinic.org/diseases-conditions/arthritis/diagnosis-treatment/drc-20350777 https://arthritisaustralia.com.au/what-is-arthritis/areas-of-the-body/hips/ https://www.medicalnewstoday.com/articles/hip-osteoarthritis-symptoms https://www.versusarthritis.org/media/22306/osteoarthritis-of-the-hip-factsheet.pdf

**Table 5:** Descriptive statistics for each readability test *Multiple modes exist. The smallest value is shown. ARI: Automated Readability Index; CLI: Coleman-Liau Index; FKGL: Flesch-Kincaid Reading Grade Level; FRES: Flesch Reading Ease Score; GFI: Gunning Fog Index; SD: standard deviation; SE: standard error; SMOG: Simple Measure of Gobbledygook

Score	FRES	FKGL	GFI	SMOG	CLI	ARI
N (Valid)	74	74	74	74	74	74
N (Missing)	0	0	0	0	0	0
Mean	56.7392	7.6216	9.2014	7.2946	13.8608	6.9095
Median	56.1500	7.3500	8.5500	6.8000	13.8500	6.8000
Mode	54.40*	6.50*	8.10	6.50	13.70	6.80
SD	8.17748	1.68655	2.09085	1.41159	1.75170	2.05734
Skewness	-0.167	1.059	1.211	1.464	0.409	1.244
SE of skewness	0.279	0.279	0.279	0.279	0.279	0.279

**Figure 2:**
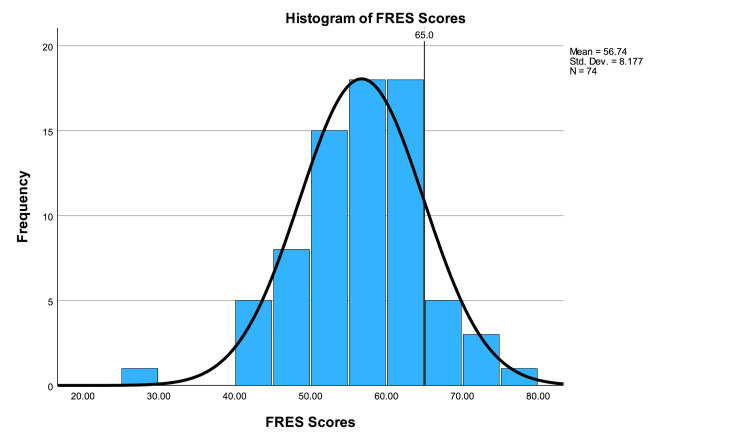
Histogram of FRES scores FRES: Flesch Reading Ease Score

**Figure 3:**
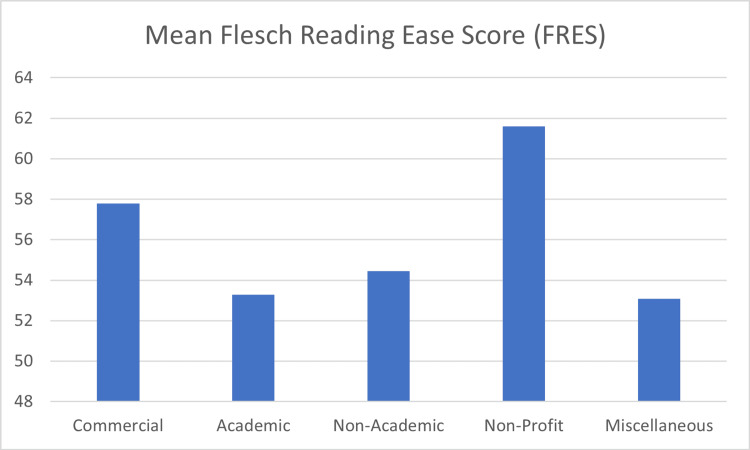
Mean Flesch Reading Ease Scores (FRES) across categories

The average FRES score was 56.74±8.18 (range 29.5-79.4), placing the data readability at grade 10 to 12 level and “fairly difficult to read”. One-sample t-testing showed that FRES scores were significantly higher than the acceptable standard (p<0.001, CI -10.2 to -6.37). Only nine (12.16%) websites had a FRES score >65. Fourteen websites (18.92%) had a FRES score <50, implying that at least a college-level education was needed to read the material. A one-way ANOVA showed a significant difference in FRES scores between groups (p=0.009). Post-hoc testing showed significant differences in scores between academic and non-profit categories (p=0.010, CI -15.17 to -1.47). FRES scores are illustrated in Figure 2. The mean readability values for FRES are presented in Figure 3.

**Figure 4:**
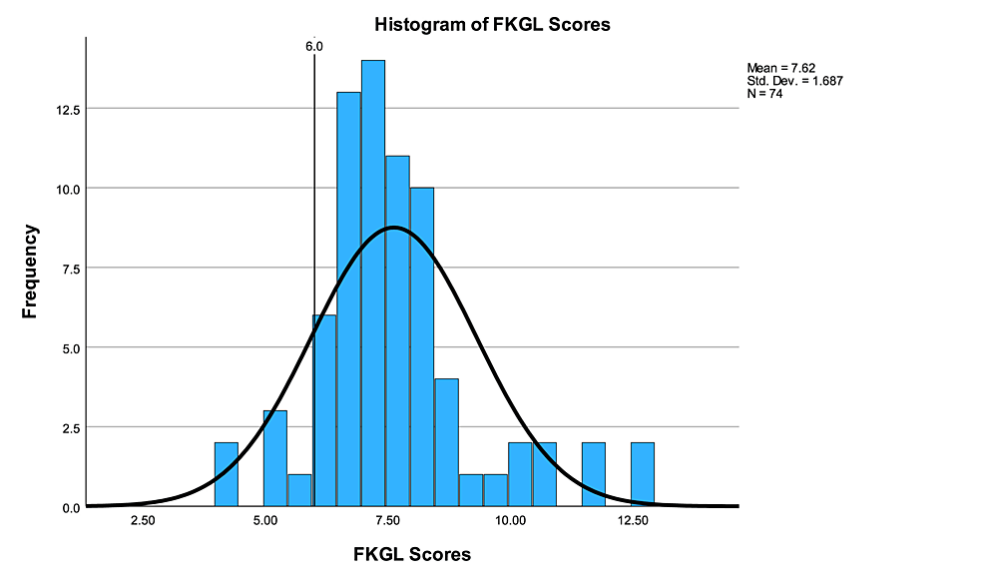
Histogram of FKGL scores FKGL: Flesch-Kincaid Reading Grade Level

The average FKGL score was 7.62±1.69 (range 4.2-12.9). One-sample t-testing showed that FKGL scores were significantly higher than the acceptable standard (p<0.001, CI 1.23 to 2.01). Only seven (9.46%) websites were written at or below a sixth-grade level according to the FKGL score. A one-way ANOVA test showed no statistically significant difference between categories (p=0.418). FKGL scores are illustrated in Figure 4.

**Figure 5:**
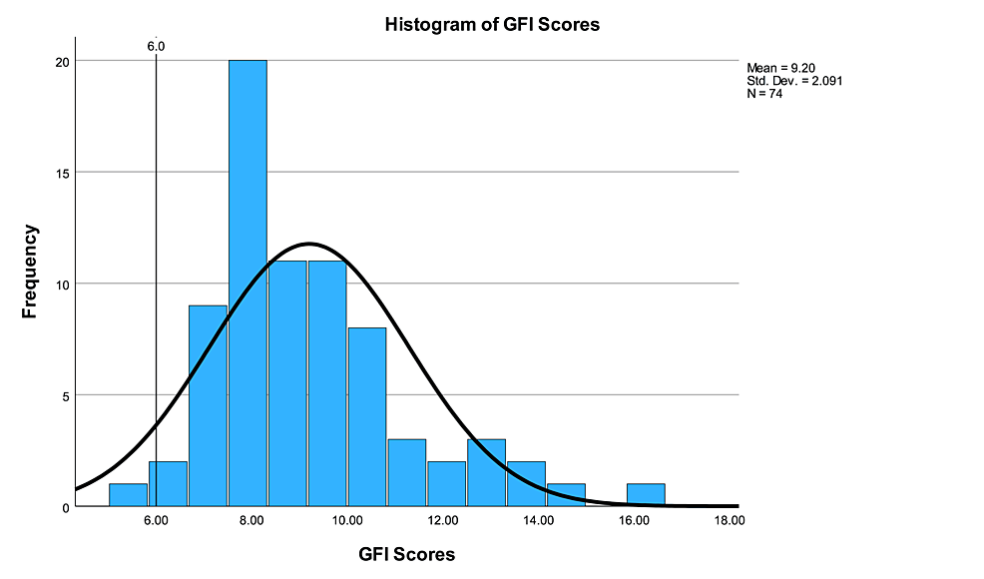
Histogram of GFI scores GFI: Gunning Fog Index

The average GFI score was 9.20±2.09 (range 5.6-16.5). One-sample t-testing showed that GFI scores were significantly higher than the acceptable standard (p<0.001, CI 2.72 to 3.69). Only one (1.35%) website was written at or below a sixth-grade level according to the GFI score. A one-way ANOVA test showed no significant difference between GFI scores based on categories (p=0.778). GFI scores are illustrated in Figure 5.

**Figure 6:**
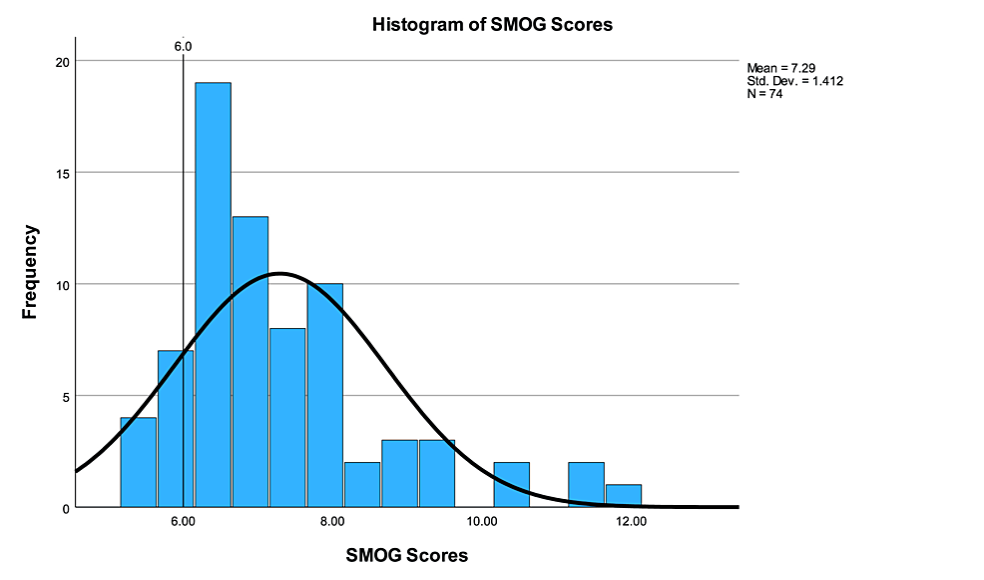
Histogram of SMOG scores SMOG: Simple Measure of Gobbledygook

The average SMOG score was 7.29±1.41 (range 5.4-12.0). One-sample t-testing showed that SMOG scores were significantly higher than the acceptable standard (p<0.001, CI 0.97 to 1.62). Only eight (10.81%) websites were written at or below a sixth-grade level according to the SMOG score. A one-way ANOVA test showed no significant difference between SMOG scores based on categories (p=0.510). SMOG scores are illustrated in Figure 6.

**Figure 7:**
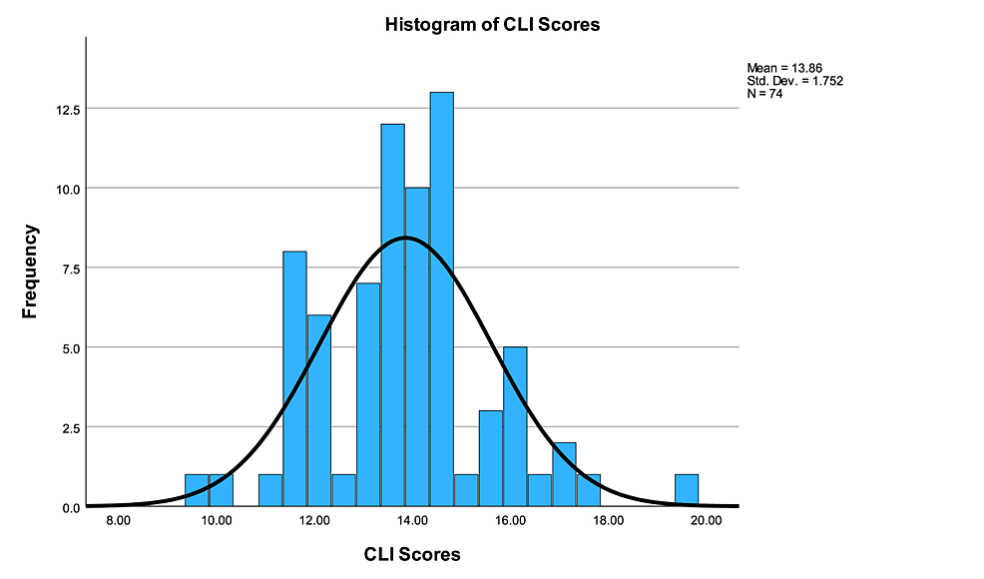
Histogram of CLI scores CLI: Coleman-Liau Index

The average CLI score was 13.86±1.75 (range 9.6-19.7). One-sample t-testing showed that CLI scores were significantly higher than the acceptable standard (p<0.001, CI 7.46 to 8.27). All 36 websites were written above a sixth-grade level according to the CLI score. A one-way ANOVA showed a significant difference in CLI scores between groups (p=0.009). Post-hoc testing showed significant differences in scores between academic and non-profit categories (p=0.008, CI 0.35 to 3.29). CLI scores are illustrated in Figure 7.

**Figure 8:**
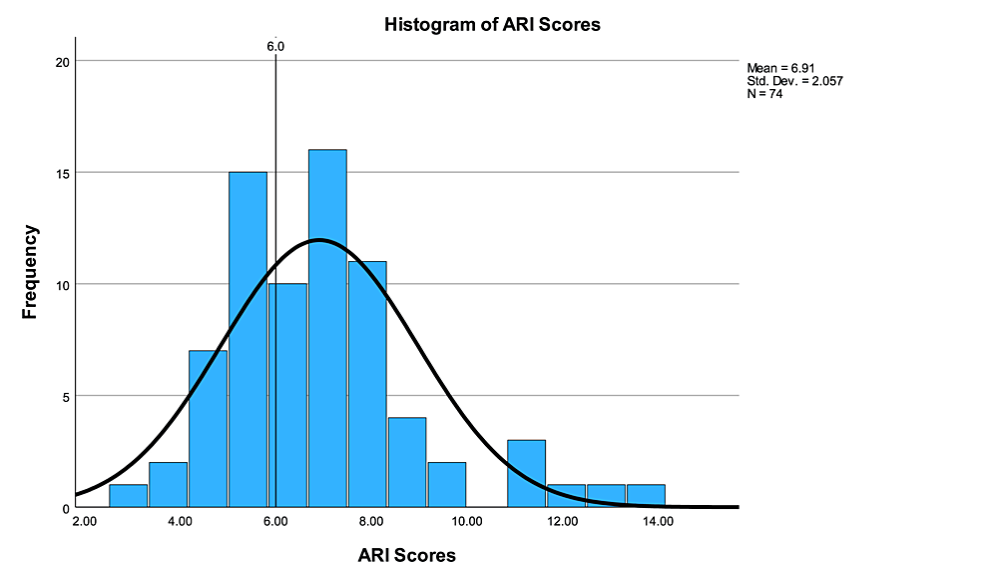
Histogram of ARI scores ARI: Automated Readability Index

**Figure 9:**
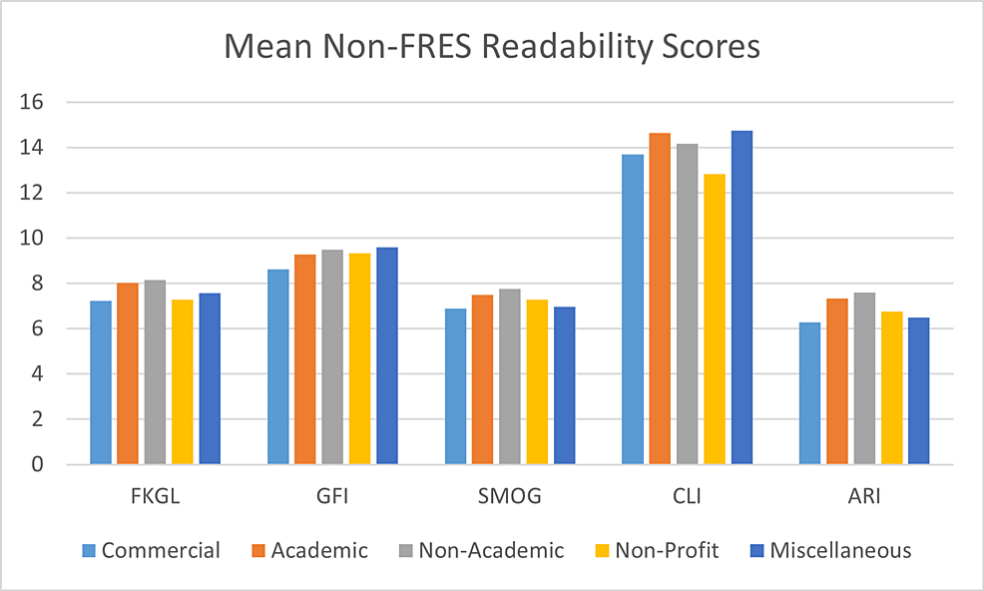
Mean non-FRES scores across categories ARI: Automated Readability Index; CLI: Coleman-Liau Index; FKGL: Flesch-Kincaid Reading Grade Level; FRES: Flesch Reading Ease Score; GFI: Gunning Fog Index; SMOG: Simple Measure of Gobbledygook

The average ARI score was 6.91±2.06 (range 3.1-14.0). One-sample t-testing showed that ARI scores were significantly higher than the acceptable standard (p<0.001, CI 0.43 to 1.39). Twenty-eight (37.84%) websites were written at or below a sixth-grade level according to the ARI score. A one-way ANOVA test showed no significant difference between ARI scores based on categories (p=0.420). ARI scores are illustrated in Figure 8. The mean readability values for non-FRES tests (FKGL, GFS, SMOG, CLI, ALI) are shown in Figure 9.

## Discussion

This study is the first to consider the readability of the online patient information resources regarding hip OA. It used six different tests to determine readability. This study found that for online resources regarding hip OA, only seven (9.46%) of the 74 websites analysed were readable at or below a sixth-grade level, according to the FKGL score, while 14 websites (18.92%) needed at least a college-level education to be read. As such, this study demonstrates majority of hip OA-related websites accessible to patients on the internet are written above the recommended sixth-grade reading level and are thus inadequate for effective patient education. This is a problem given how important the Internet is to the provision of patient education. In 2003, the number of health-related searches on the internet was at least 6.75 million per day [27]. Almost 20 years later, internet penetrance is expected to reach 97% with 90% of patients relying on the internet as their primary source of health-related information [6].

In the field of Orthopaedic Surgery, Daraz et al. (2018) found that the mean FKGL score for online resources regarding Orthopaedics was slightly above 10 [10]. This is almost three grades higher than the FKGL scores of hip OA-related websites. Specific to OA, a study of online information regarding OA carried out in 2019 found a mean FRES score of 51.4, a mean FKGL score of 7.8, and a mean GFI score of 9.0 [12]. Comparing these results with our findings, the readability of online patient information resources regarding hip OA has improved in the last five years compared to the readability of resources regarding OA in general. The median FRES and FKGL scores of hand OA-related websites were found to be 52.20 and 10.30 respectively [17]. The median FRES and FKGL scores of knee OA-related websites were found to be 53 and eight respectively [16]. Hip OA-related websites are thus more readable than hand and knee OA-related websites. Therefore, although the recommended standards have yet to be met, the improvement in the readability of hip OA-related internet resources compared to previous studies in the literature is promising.

The problem regarding online healthcare resources being written above recommended patient reading levels is prevalent in other disciplines across medicine and surgery, from Orthopaedic Surgery [6,14,25] to Oncology [23] and Rheumatology [28]. Regarding whether there were any differences in readability between website categories, one-way ANOVA testing found statistically significant differences between different website types as per the FRES (p=0.009) and CLI (p=0.009) scores while post hoc testing showed a significant difference between academic and non-profit categories for FRES scores (p=0.010, CI -15.17 to -1.47) and CLI scores (p=0.008, CI 0.35 to 3.29). This demonstrated that the readability of online information regarding hip OA differed depending on the person or organisation providing said information. While academic websites may be targeted at other clinicians, commercial, government, and nonprofit websites that are aimed at patients have an even greater responsibility to simplify their content for readers [28]. Steps that website creators can take include using less complex language and less jargon [28], supplementing text with videos and images [25], considering patient input and preferences [11], and utilising free online tools such as WebFX to evaluate using the readability of written information before publishing it online [21].

This study was not without limitations. Regarding the search, the first 30 results of each search were screened which may have resulted in relevant websites beyond the first 30 results being excluded. Other less popular search engines like Yahoo were not utilised which may have produced different search results. Materials on the internet also change from day to day and the top search results may differ depending on a user’s cookies or location. Regarding the analysis of websites, the WebFX online software tool does not consider illustrations and videos that may have been useful in complementing text to enhance patient understanding. The readability tools used to assess websites were not originally designed to assess health literature and do not use health content in their validation [29]. Furthermore, these tools determine readability according to the number of characters or syllables per word without considering their meanings which can lead to an inaccurate representation of how difficult a word is to understand. There are also more readability and comprehension instruments than the six utilized in this review such as the Dale-Chall readability formula and the Fry readability formula [29,30] which may have produced different results.

## Conclusions

Evaluating the readability of current online resources regarding hip OA using six different validated algorithms for readability found that most websites are written above the recommended reading grade level. Steps must be taken by providers of these online resources to simplify content and avoid complex language and jargon. Doing so will improve readability and patient understanding of the material which can ultimately improve treatment outcomes and patient satisfaction.
